# Characterization, commissioning, and clinical evaluation of a commercial BeO optically stimulated luminescence (OSL) system

**DOI:** 10.1002/acm2.70057

**Published:** 2025-02-22

**Authors:** Joseph P. Kowalski, Brett G. Erickson, Qiuwen Wu, Xinyi Li, Sua Yoo

**Affiliations:** ^1^ Department of Radiation Oncology Duke University Medical Center Durham North Carolina USA; ^2^ Department of Radiation Oncology Mayo Clinic Rochester Minnesota USA

**Keywords:** beryllium oxide, in vivo dosimetry, optically stimulated luminescent dosimeter

## Abstract

This article investigates the performance of a commercial BeO optically stimulated luminescent (OSL) dosimetry system (myOSLchip, RadPro GmbH International, Remscheid, Germany) through the application of the commissioning framework for luminescent dosimeters as described in the American Association of Physicists in Medicine Task Group 191 (AAPM TG191) report. Initial clinical experiences and dosimetric results are also presented. The following properties of the system were characterized: linearity correction factors ranged from ‐0.5% to +3% for dose levels spanning 0.1 to 20 Gy. Beam quality correction factors (relative to 6 MV) ranged from ‐4.5% (2.5FFF) to +4.5% (15MV) for photon beams and +1.9% (6 MeV) to +4.3% (20 MeV) for electron beams. An average (µ) signal loss per reading of ‐2.13% ± 0.20% was measured, however greater signal loss was observed in the first reading (µ = ‐2.6% ± 0.46%). An initial decline in individual element sensitivity relative to baseline was observed from 0–15 Gy cumulative dose (µ = ‐1.98% ± 0.55%), with negligible further deterioration from 15–32 Gy (µ = ‐2.38% ± 0.85%). Post‐irradiation, there was a transient OSL signal which faded with a half‐life of 1.8 min; this signal enhancement was +5% at 5 min post‐irradiation and +1% at 15 min relative to 24 h. Dosimeter response was not dependent on average dose rate in the range of 100–2500 MU/min. With respect to clinical testing, equal or superior performance compared with aluminum oxide OSLs (nanoDots) is shown for a range of clinical techniques and modalities including TSET, TBI, en‐face electrons, and pacemaker/out‐of‐field measurements. The feasibility of myOSLchip to serve as a primary clinical in vivo dosimetry system and direct replacement for Landauer's microStar system is demonstrated.

## 1 INTRODUCTION

Optically stimulated luminescence is a property of certain insulators and semiconductors that is observed following exposure to ionizing radiation. During irradiation, a signal is stored within the material in the form of electrons caught in traps. These traps are created through the controlled introduction of crystal defects and possess electrical potentials below that of the material's conduction band. When the material is stimulated with photons possessing energies in excess of the trap potential, electrons are promoted to the conduction band after which they migrate to recombination centers and undergo de‐excitation through photon emission.^1^ Emitted photon wavelengths differ from those used for stimulation.

Numerous materials have been evaluated^2^, ^3^, ^4^ in addition to aluminum oxide, including beryllium oxide and magnesium oxides; however, within the medical dosimetry community, carbon‐doped aluminum oxide (Al_2_O_3_:C) stands out both in terms of the degree to which it has been studied^5^ and its breadth of adoption. Within the United States, the primary commercial vendor was Landauer Inc. which offered both dosimeters (nanoDot) and readers (microStar, microStar ii) for in vivo dosimetry use. These systems were utilized at this institution—Duke University Medical Center—from May 2011 onward.

OSL dosimeters possess a number of advantageous characteristics including high sensitivity, linearity of response, reusability, ease of readout, long‐term signal stability, and temperature independence over a wide range.^5^, ^6^ Many of these features are particularly beneficial in multi‐site, high‐volume settings. As with any technology, there are also limitations. With respect to aluminum oxide OSLs, several studies have reported element sensitivity changes with accumulated dose.^7^, ^8^, ^9^ Additionally, substantial non‐linear response to dose at levels above 2 Gy has also been observed.^2^, ^6^, ^10^ However, it has also been demonstrated that when these dependencies and behaviors are accurately characterized and properly accounted for, clinically acceptable accuracy and precision is obtainable.^11^

In the second half of 2023, Landauer released a series of FDA recall notices covering both their dosimeters and the microStar ii reader. In these notices, they also announced their intention to end support for their medical dosimetry program, forcing users to turn elsewhere for in‐vivo dosimetry solutions. Surprisingly, the number of commercially available OSL systems is small, but other offerings are available,^12^ including the myOSLchip system (RadPro/Freiberg Instruments GmbH, Heidelberg, Germany) which is focused on BeO.

The American Association of Physicists in Medicine (AAPM) Task Group (TG) 191 presented a framework for characterization and commissioning of luminescent dosimetry systems. In this paper, the results of applying this methodology to the myOSLchip system are presented alongside initial clinical findings.

## 2 MATERIALS AND METHODS

In this section, the basis of operation of the RadPro myOSLchip system and its manufacturer‐specified operating characteristics and limits are described. Subsequently, the OSL dose calculation formalism described in the AAPM TG191 report is discussed. Finally, a detailed description of the methodology used for the determination of dose correction factors and an overview of the clinical commissioning process are presented.

### 2.1 Operational fundamentals of the myOSLchip system

**FIGURE 1:**
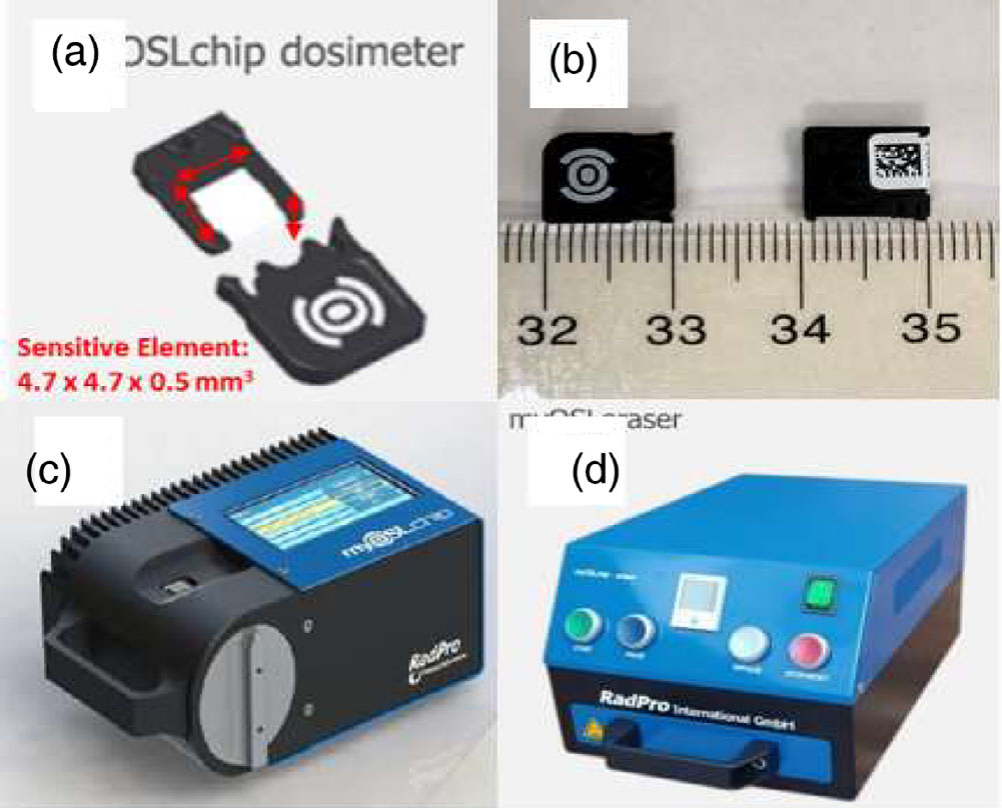
The myOSLchip system: (a) the myOSL BeO dosimeter with sensitive element dimensions; (b) a side‐by‐side comparison between the BeO OSL and a nanoDot; (c) the myOSLchip reader; (d) the myOSLchip standalone bleaching unit.

The myOSLchip in‐vivo patient dosimetry system consists of a portable reader, a dedicated bleaching unit, and a set of reusable BeO chips (Figure 1). The system was designed and manufactured by RadPro International GmbH in collaboration with Freiberg Instruments.

Each chip consists of a square‐faced sensitive element measuring 4.7 × 4.7 × 0.5 mm^3^ (L x W x H) encased in an acrylonitrile butadiene styrene (ABS) shell. The beryllium oxide (Z_eff_ = 7.2) used for these chips is sourced from Materion Corporation (trade name: Thermalox 995) and has a reported purity of 99.5%. Per the manufacturer, myOSLchip elements are reusable, have an accumulated dose limit of 30 Gy, and demonstrate linear response with dose up to 10 Gy. Note that this cumulative dose limit is in addition to a pre‐conditioning dose of 15 Gy delivered by the vendor using a ^90^Sr/^90^Y irradiator to all of its high dose/therapeutic dosimeters (RadPro, personal correspondence). This stabilizing dose is necessary due to the large sensitivity changes exhibited by new, unirradiated BeO chips. Broadhead et al.^13^ demonstrated that while this initial change can exceed ‐40% relative to baseline, chip sensitivity subsequently stabilizes at cumulative dose levels between 6 and 10 Gy. While this dose is a part of each chip's irradiation history, cumulative dose values referenced in this paper refer exclusively to in‐clinic irradiations and disregard this stabilization dose.

The reader itself is designed primarily for operation as a fixed measurement station (connected to a PC) running the OSLDosimetry software; however, it is capable of limited standalone operations. During readout, a single, filtered LED (100 mW) with a central wavelength of 460 nm uniformly illuminates the entire sensitive element for 0.2s while signal collection proceeds simultaneously.^14^ The stimulated emissions—noted in a previous study^15^ to predominantly arise from two bands centered on 310 nm and 370 nm (primary)—are passed through a filtering element and onto an amplified photosensor. The myOSLchip reader adjusts some aspects of signal processing based on dose level with a transition point set around 12 Gy. In contrast to the microStar readers, photosensor gain is adjusted instead of illumination strength. In addition to read operations, the reader also features an on‐board erase capability served by separate high‐power LED (1 W).

The bleaching unit, which can service up to 48 chips simultaneously, utilizes an array of 5‐watt light‐emitting diodes (LEDs) with a peak wavelength of 480 nm to erase residual chip signal. By default, the bleacher is set to run for the maximum allowable interval (120 min), however this can be reduced based on user requirements.

### 2.2 The TG‐191 dose calculation and commissioning formalisms

The report of AAPM Task Group 191 presents a framework for calculation of luminescent dosimeter (LD) dose that mirrors TG 51. That is, dose to water is obtained through the multiplication of a corrected reading (M_corr_) with a set of detector‐specific correction factors (k_x_) and a system calibration coefficient (N_D,w_).

(1) $$D_{w}=M_{corr}*N_{D,w}*k_{F}*k_{L}*k_{Q}*k_{\theta }$$

The corrected reading is obtained through multiplication of a dosimeter's background‐adjusted signal with its individual element sensitivity (k_s,i_). Other correction factors account for response variation relative to a reference condition due to fading (k_F_), linearity (k_L_), beam quality (k_Q_), and angular dependence (k_θ_).

TG‐191 further describes two main approaches to system commissioning: one focused on high accuracy, and the other on high efficiency. Maximization of accuracy is achieved through measurement and application of a complete set of correction factors, including individual element sensitivities. Alternatively, the high‐efficiency approach sacrifices some accuracy in exchange for a more streamlined and less burdensome commissioning and operational workload. Measurement and application of correction factors is minimized. Instead, acceptable limits for correction factors and individual element sensitivities are set by the end user and only LDs that satisfy these criteria are used within the clinic.

In this paper, we describe our initial experience performing a high accuracy‐oriented commissioning process for the myOSLchip system including correction factor measurement and clinical validation.

### 2.3 System configuration

As previously mentioned, the myOSLchip system can be configured for portable or stationary operations. The latter configuration was employed at our institution and requires installation of a proprietary software on a PC for interfacing with the device. All discussions herein pertain to that specific configuration. System setup and configuration for stationary operations is a 4‐step process: Registration, Device Calibration, Dosimeter Calibration, System Calibration. Each step is subsequently covered in detail.

#### 2.3.1 Registration

Dosimeter registration in the software database is required for all operations aside from erasure. During this step, the user assigns a dosimeter type (Field or Calibration) and an internal workflow (Read Only, or Read‐Erase‐Read). It should be mentioned that calibration dosimeters are required for system configuration steps, however there are no other functional differences with Field dosimeters.

During the registration process, the reader will automatically verify the dosimeter QR code and record an initial background reading. Once complete, the user proceeds to Device Calibration.

#### 2.3.2 Device calibration

This step establishes the device sensitivity factor (DSF). The user irradiates a set of calibration dosimeters to a known dose. For initial system setup, all individual dosimeter sensitivities are assumed to be 1. Subsequent device calibrations utilize measured element sensitivities. The DSF is calculated as a simple average of the set of signal/dose ratios and has units of [counts/unit dose]. At our clinic, a set of 13 dosimeters was used for completion of this step. These chips were irradiated under the reference conditions described in 2.D and allowed to rest for 24 h prior to readout.

#### 2.3.3 Dosimeter calibration

In this step, individual element sensitivity factors (ESF) are established. Once again, dosimeters are irradiated to a known dose and readout by the system. The individual element sensitivity factor is calculated by the system such that:

(2) $$ESF=\frac{DSF\left[\frac{counts}{dose}\right]*Calibration\;Dose\left[dose\right]}{M_{i}\left[counts\right]}$$

#### 2.3.4 System calibration

The system calibration step was originally implemented for centers that did not possess the means to relate a given local exposure to a dosimetric standard maintained by an accredited calibration laboratory and thus could not independently establish a local exposure/dose relationship. It allows for establishment of an additional correction factor based on the comparison between locally irradiated dosimeters and dosimeters irradiated against a dosimetric standard to a known dose.

For institutions with calibrated ion chambers, this step is not explicitly necessary, however system setup cannot be finished without completion of this step. In order to satisfy this software requirement, we re‐irradiated the same set of calibration chips used for Device Calibration under reference conditions as described below in 2.D and read them out after a 24‐h delay. For institutions performing this step pro forma, a system calibration factor very close to 1 would be expected.

#### 2.3.5 System dose calculations

When a measurement is performed, the process starts with a single read operation. This counts value is automatically corrected for background ($M_{corr})$, divided by the device sensitivity factor, and multiplied with the individual element sensitivity ($k_{s,i})$ and the system calibration factor (SCF). Finally, the result is multiplied with an energy correction factor and a dose result is presented. If user‐defined energy correction factors are not entered in the system software, a value of 1.0 is used. In equation form:

(3) $$Dose=\frac{M_{corr}\left[cts\right]*k_{s,i}*SCF*k_{Q}}{DSF\left[\frac{cts}{dose\;unit}\right]}$$

In the present software version, if the user desires to apply additional corrections to the dose reading, these must be performed manually outside of the software.

### 2.4 Correction factor measurement

Correction factors are calculated with respect to a reference condition. All irradiations were performed using a Varian TrueBeam linear accelerator. While choice of reference condition is arbitrary, our selection was made with a focus on clinical applicability, minimization of electron contamination, and maximization of chip throughput. Reference irradiations were performed with a 6 MV beam at a depth of 10 cm, with a field size of 20 × 20 cm^2^, and an SSD of 100 cm. Under these conditions, the corresponding percent depth dose (PDD) is 69.5%. Additionally, a reference dose level of 2 Gy was used, and all chips were allowed to rest for 24 h prior to readout. Prior to irradiation, machine output was verified daily in solid water by ionization chamber (IBA FC65‐P, farmer‐type, 0.6 cc) for each energy used. The same solid water pieces and stacking order were used throughout the commissioning process.

**FIGURE 2:**
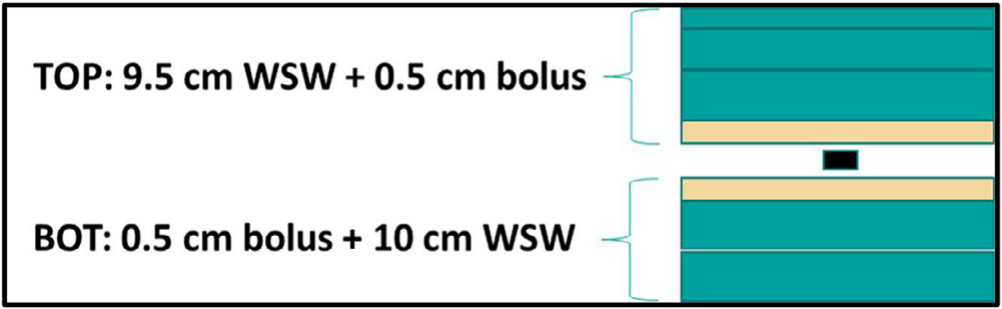
Graphical depiction of the solid water/bolus arrangement for reference irradiation measurements.

Chips were irradiated simultaneously in batches of up to 20. Care was taken to ensure that all chips were positioned within 3 cm of central axis, corresponding to a maximum dose difference of 0.9% for 6 MV. Dosimeters were positioned between two 0.5 cm pieces of Superflab bolus to reduce mechanical stress on the chips and minimize air gaps as depicted in Figure 2. 10 cm of solid water was placed below the chips to ensure scatter equilibrium, and 9.5 cm of solid water was placed above the chips to achieve the desired radiological depth. Additional details relevant to individual correction factor measurements are discussed below.

#### 2.4.1 Element sensitivity correction factor ($k_{s,i}$)

The element sensitivity correction factor ($k_{s,i}$) quantifies the variability in single chip response with respect to the entire population. Measurements were performed using the reference setup described in 2.D, and factors were calculated in accordance with the TG‐191 definition for batch calibrations as the ratio of the batch mean response ($\bar{M}$) to the individual element response ($M_{i})$:

(4) $$k_{s,i}=\frac{\bar{M}}{M_{i}}$$

Long‐term stability of individual element sensitivity was assessed in relative and absolute terms. Relative response variation with accumulated dose was investigated by comparing population statistics—derived on a per irradiation basis—of the same set of 60 chips from 3 distinct irradiations performed at t = 0 (baseline), t+36d, and t+131d. Absolute changes in chip sensitivity with cumulative dose were assessed by grouping chips based on cumulative dose and analyzing changes in response on an intra‐chip basis.

Additionally, a comparison was made between sensitivity correction factors and those determined by the vendor to evaluate the influence of irradiation source and dose level on sensitivity determination (the vendor sensitivity determination is performed utilizing a custom 33 MBq ^90^Sr/^90^Y irradiator and a very low dose on the order of 0.01 Gy).

#### 2.4.2 Fading correction (k_F_)

The fading correction factor accounts for signal loss within the OSL due to spontaneous clearance of low‐energy electron traps following irradiation. TG‐191 defines the fading correction in terms of the following ratio of doses ($D_{x})$ and their associated signal counts $\left(M_{x}\right)$:

(5) $$k_{F}\left(t\right)=\frac{\frac{D_{exp}}{M_{exp}\left(t_{exp}\right)}}{\frac{D_{ref}}{M_{ref}\left(t_{ref}=24\;h\right)}}$$

where “exp” refers to the experimental condition and “ref” refers to the reference condition. Fading corrections were determined via a two‐step process. Chips were irradiated and readout as described in 2.D for both the experimental and reference measurements. All dosimeters were then bleached and re‐irradiated to 2 Gy. M_exp_ was then obtained by varying the readout time delay relative to the reference (24 h). Due to the need for timely results in clinical practice, the primary focus of the initial fading analysis centered on the first 24 h post‐irradiation.

#### 2.4.3 Beam quality correction ($k_{Q}$)

The beam quality correction factor ($k_{Q}$) accounts for variation in dosimeter response due to intrinsic material property differences and medium‐dependent factors. As described in TG‐191, intrinsic energy dependence is a property of the dosimeter material itself and accounts for the impact of differing microscopic dose distributions on material trapping and recombination mechanisms within the luminescent dosimeter. The second—medium‐dependent energy dependence—accounts for the impact of differing attenuation coefficients and stopping‐power ratios between the detector and the reference medium.

Clinically, there is no need to separate these effects and, in this paper, composite beam quality correction factors were determined. TG‐191 expresses this factor as:

(6) $$k_{Q}=\frac{\frac{D_{exp}}{M_{exp}\left(Q_{exp}\right)}}{\frac{D_{ref}}{M_{ref}\left(Q_{ref}=6\;MV\right)}}$$

6 MV was used as the reference energy and irradiation conditions were as described in 2.D with exceptions specified below. The reference signal used was the mean signal (${\bar{M}}_{ref}$) of a given chip from all reference condition irradiations. Flattened photon beam (10 MV, 15 MV) irradiations were carried out in batches of up to 10 chips with a maximum displacement from central axis of 2 cm, corresponding to a maximum dose error of 0.5% for 10 MV and 1.4% for 15 MV at a depth of 10 cm. Due to rapid changes in the radial profiles of flattening filter free (FFF) beams with distance from CAX, these irradiations were performed one chip at a time. 400 MU/min was used as the reference dose rate for 10 FFF due to lack of a 600 MU/min option. With respect to 2.5FFF, historical data availability and dose rate options necessitated the use of a 10 × 10 cm^2^ field size and a dose rate of 60 MU/min.

Electron measurements were performed using an A15 applicator along with the standard, vendor‐supplied open inserts for 6/9/12/16/20 MeV. All electron irradiations were carried out at d_max_ (9e: 2.0 cm, 12e: 2.8 cm, 16e: 3.5 cm, 20e: 3.0 cm) with the exception of 6 MeV (d = 1.3 cm, PDD = 99.66%) due to our limited selection of solid water thicknesses.

#### 2.4.4 Average dose rate dependence

The impact of varying average dose rate was evaluated using flattened (6 MV) and flattening filter free photon beams (6/10 FFF), and 6 HDTSe at a depth of 1.3 cm (FS = 36 × 36 cm^2^ PDD = 99.58%). Measurement data for this test was acquired using single‐chip irradiations only. 6 FFF comparisons were made between dose rates of 600 and 1400 MU/min. 10 FFF comparisons were made between 400 and 2400 MU/min, and 6 MV comparisons were made using dose raters of 100 and 600 MU/min. Due to the availability of a single dose rate for 6 HDTSe (2500 MU/min), these measurements were compared directly with 6e readings at 600 MU/min given the near‐equivalent beam qualities. Results were calculated as the ratio of experimental to reference counts. All other irradiation parameters were as described in 2.D.

#### 2.4.5 Depletion

Signal loss per reading (depletion) was determined through repeated comparison of the current measured signal with the preceding value for a given chip. The impacts of dose, energy, and measurement frequency on this value were evaluated. All testing was performed 24 h post‐irradiation to mitigate the influence of fading on the measurement.

#### 2.4.6 Linearity (k_L_)

The linearity correction factor, (k_L_) accounts for dosimeter sensitivity changes as a function of dose. In the range of doses used for therapeutic purposes, this response is known to be supralinear. Dosimeter response as a function of irradiated dose was calculated per TG‐191 as the ratio of the experimental counts per unit dose to the equivalent reference quantity. That is:

(7) $$k_{L}\left(D\right)=\frac{\frac{D_{exp}}{M_{exp}\left(D_{exp}\right)}}{\frac{D_{ref}}{M_{ref}\left(D_{ref}=2\;Gy\right)}}$$

Reference measurements were carried out using the reference setup as described in 2.D and 2 Gy was used as the reference dose level. The reference signal used was the mean signal (${\bar{M}}_{ref}$) for a given chip across all reference condition irradiations. For experimental measurements, all parameters were held constant with the exception of dose. Dose levels ranging from 0.1–20 Gy were evaluated.

#### 2.4.7 Angular dependence

The angular dependence correction factor (k_θ_) accounts the variation in dosimeter response arising from non‐orthogonal beam incidence. Per TG191, this characteristic is a property of the dosimeter itself that is typically specified in terms of relative response to an en face orientation (θ = 0°):

(8a) $$k_{\theta }=\frac{M_{i,\theta_{exp}}}{M_{i,\theta =0}}$$

This factor was assessed in the following manner. Three chips were selected for each angle. A reference measurement (setup per 2.D.) was first performed for each chip. Dosimeters were subsequently bleached and prepared for re‐irradiation at one of four experimental angles {0°, 30°, 60°, 90°}. Angular irradiations were performed by wrapping the chip in bolus and placing the dosimeter at the center of the innermost CTDI phantom (diameter = 10 cm) which itself was placed at isocenter. After dosimeter loading, the phantom was then rotated to the appropriate experimental angle using angular markings on the exterior of the cylinder. Care was taken to wrap each chip using the same bolus pieces in the same orientation each time. Each angular irradiation was performed under the following conditions: E = 6MV, SSD = 95 cm, d = 5 cm, FS = 15 × 15 cm^2^, MU = 200, G = 0°. Correction factors were calculated by first normalizing all measurements to their own reference count value, and then averaging response by angle. Finally, angular dependence was assessed by dividing the normalized average for a given angle by the normalized average for 0°:

(8b) $$k_{\theta }=\bar{\sum_{i=1}^{i=3}\frac{M_{i,\theta }}{M_{i,ref}}}/\bar{\sum_{i=1}^{i=3}\frac{M_{i,\theta =0}}{M_{i,ref}}}$$

#### 2.4.8 Clinical validation

Clinical validation was accomplished through a mix of direct, real‐time comparisons against our established in‐vivo OSL dosimetry standard (Landauer microStar ii / nanoDot) and comparisons of standalone beryllium oxide OSL measurements against commissioning values and historical nanoDot readings for similar clinical scenarios.

Measurements were performed for a variety of “standard” electron and photon techniques including en face electron treatments, pacemaker measurements, and isocentric photon treatments at various energies. Special procedure measurements for Total Skin Irradiation (TSI, standing and recumbent) and Total Body Irradiation (TBI) were also performed and compared.

Pacemaker measurements were performed at varying distances from the field edge. In accordance with our protocol, all OSLs were taped directly above the pacemaker and underneath 1 cm of bolus. Toroidal magnets were then placed atop the bolus if directed/required by Cardiology.

All chip readouts were performed 30 to 60 min post‐irradiation to simulate an actual clinical workflow. In addition, measured beam quality correction factors (Figure 5) were applied to all BeO measurements.

Thorough testing and characterization of nanoDot batches used in this study was performed to verify accuracy and maintain the integrity of measurement comparisons.

## 3 RESULTS

### 3.1 Element sensitivity correction factor ($k_{s,i}$)

**FIGURE 3:**
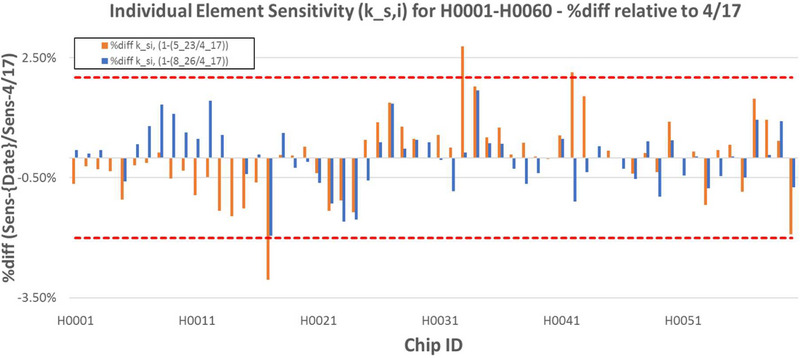
Individual element (H0001‐H0060) sensitivity percent differences relative to baseline (4/17/2024). Compared values were calculated in accordance with Equation 3 on a per‐irradiation basis.

Element sensitivity correction factors demonstrated good stability over time. Three irradiations for a 60‐chip set were performed on 4/17/2024 (baseline), 5/23 (t+31d), and 8/26 (t+136d). Element sensitivity correction factors were computed for the chip set following each irradiation. On a relative basis, 95% (57/60) of chips demonstrated a change from baseline of < 2% following the second irradiation, and 100% of chips met this threshold following the third irradiation as shown in Figure 3. A snapshot of chip population statistics (post‐irradiation) at the time of each irradiation event is shown in Table 1.

**TABLE 1:** Population statistics for chips H0001‐H0060 used for individual element sensitivity variation with accumulated dose. Data from 4/17/2024 used as baseline.

Irradiation date	Mean counts	STDEV	cts/cGy	Min Cum Dose [cGy]	Max Cum Dose [cGy]	Median dose [cGy]
4/17/2024	38380	2509.7	191.9	200	600	—
5/23/2024	37764	2549.1	188.8	800	2700	1400
8/26/2024	37565	2480.9	187.8	1300	3200	2000

**FIGURE 4:**
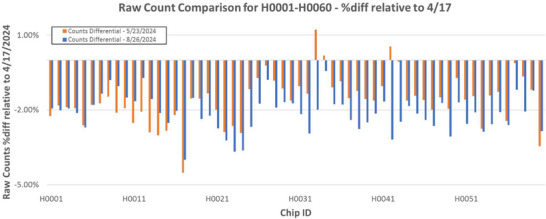
Variation in individual element (H0001‐H0060) raw counts. Percent differences are relative to baseline counts as measured on 4/17/2024.

In terms of absolute dosimeter performance, Figure 4 provides an overview of variation in raw counts on a per‐dosimeter basis relative to baseline. The average chip response relative to baseline was ‐1.61% ± 0.97% {‐1.58%} and ‐2.13% ± 0.72% {‐2.09%} for 5/23 and 8/26 respectively. Additionally, no significant difference in baseline sensitivity shift was observed when comparing chip groups with varying cumulative doses (as of 8/26) as shown in Table 2.

**TABLE 2:** Sensitivity shift from baseline as a function of cumulative dose. This data was generated through comparison of 8/26/2024 values against those measured on 4/17/2024.

Dose range [Gy]	Sample size	Mean (µ) Sensitivity shift relative to baseline	Standard deviation (1σ)
10–15	20	−1.98%	0.55%
15–20	17	−2.17%	0.72%
20–25	9	−1.99%	0.86%
25–32	14	−2.37%	0.85%

Of note, the system vendor (RadPro) offers a sensitivity determination service which works similarly to the “pre‐screening” option formerly available for nanoDots from Landauer. A comparison was made between our measured baseline sensitivities and those provided by the vendor. Results were generally in good agreement with 95% of chips demonstrating a relative deviation of less than ± 3% (maximum relative deviation of ‐4.5%).

### 3.2 Beam quality correction factor ($k_{Q}$)

**FIGURE 5:**
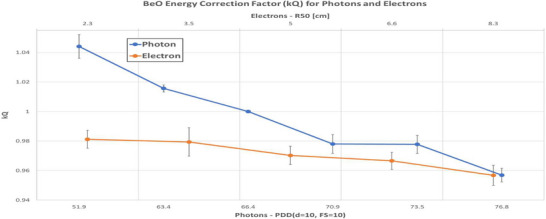
Beam quality correction factors for BeO (1 standard deviation) relative to 6 MV. Photon measurements were performed at a depth of 10 cm, while electron measurements were performed at dmax.

Beam quality correction factors were measured for both therapeutic photon and electron beams using Varian TrueBeam linear accelerators. In the TG‐191 formalism, k_Q_ > 1 indicates an under‐response relative to the reference while k_Q_ < 1 indicates over‐response. Relative response to 6 MV correlated strongly with relative beam quality for both electrons and photons. Photon beam energy correction factors ranged in magnitude from 1.044 ± 0.0080 (2.5FFF) to 0.9568 ± 0.0046 (15 MV). For electron energies, correction factor values ranged from 0.9812 ± 0.0061 (6 MeV) to 0.9567 ± 0.0068 (20 MeV) as shown in Figure 5. Listed ranges are for one standard deviation.

### 3.3 Fading correction

**FIGURE 6:**
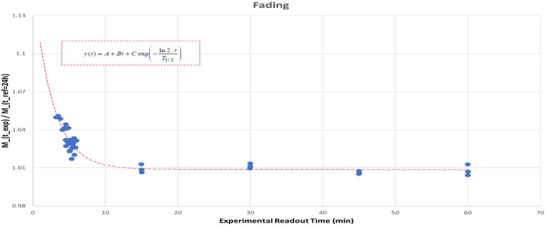
Measured fading behavior for BeO relative to +24 h post‐irradiation with linear + exponential decay best fit line (red‐dashes). Fitting equation shown in upper‐left.

To prepare the system for clinical use, fading correction analysis was focused on the first 24 h post‐irradiation (Figure 6). The t+24 h datapoint was used for normalization. Rapid signal falloff within the first 10–15 min post‐irradiation was observed, consistent with prior OSL studies focused on aluminum oxide dosimeters.^2^, ^10^ From 15–60 min, a much more gradual decline in signal of from +1.0% to +0.8% was observed. For characterization of the signal deterioration, an exponential plus linear function of the form employed by Mrčela et al. was fitted to the data with fitting parameters A, B, C, and T_1/2_ using the GRG Nonlinear Solving Method within Excel.

This fitting yielded a T_1/2_ parameter of 1.857 m, slightly longer than, but of comparable magnitude to previously reported values for aluminum oxide‐based chips of 0.75 to 1.44 m^2^.^10^

### 3.4 Dose linearity correction

**FIGURE 7:**
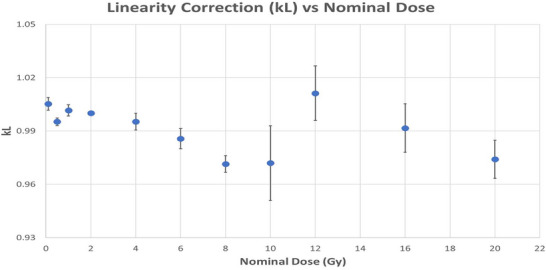
Linearity correction factors for BeO (error bars: 1 standard deviation) calculated in accordance with Equation 6.

Dose linearity was assessed across the dose range of 0.1–20 Gy. In general, a trend of slowly increasing supralinear response (k_L_ < 1, per TG‐191) with dose was observed, consistent with prior OSL studies of BeO and Al_2_O_3_:C,^10^, ^13^ with a caveat. When the myOSLchip reader detects a high dose reading (≥ 2.25 × 10^5^ counts, or ∼12 Gy), photosensor gain is reduced by a factor of 4.16 (RadPro, personal communication, 2024). A 4% shift in system response between 10 Gy (3% supralinear) and 12 Gy (1% sub‐linear) is observed at this dose level relative to 2 Gy as shown in Figure 7. From 12–20 Gy, chip response returns to the familiar trend of increasing supralinear response with dose. The net result of this system behavior is a maximum linearity effect of ∼ 3% across the full extent of the explored dose range.

### 3.5 Depletion

**FIGURE 8:**
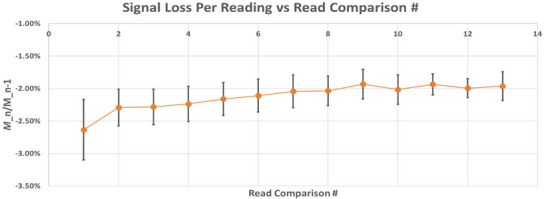
Averaged signal loss per reading values for BeO (error bars: 1 standard deviation). Signal loss estimated by a comparison of successive read raw counts.

An average signal depletion of −2.13% ± 0.20 % per read was measured for the myOSLchip system out to 14 successive reads; however, a larger initial signal loss was also observed (−2.6% ± 0.46%). Subsequent comparisons converged to the mean value cited above. No significant differences were observed in terms of signal depletion with respect to average dose rate, readout frequency, energy, or total dose. Data for average signal loss per read for the first 13 comparisons is shown in Figure 8.

### 3.6 Phantom and patient measurements

Verification of measured correction factors and overall system function was validated with a series of phantom and clinical measurements (Table 3). In‐vivo measurements were performed for 5 lateral Total Body Irradiation (TBI) patients, 6 en‐face electron patients, and 2 Total Skin Electron Therapy (TSET) cases. Additionally, out‐of‐field doses were measured for 7 patients with pacemakers/ICDs. TSET data was acquired for one standing technique case (modified Stanford technique^16^) and for one recumbent/laydown technique patient.^17^ Percentage differences between BeO and reference values (e.g., nanoDot, commissioning measurements) were calculated as:

(9) $$Percentage\;Difference=\left(\frac{BeO\;Dose}{Reference\;Dose}\right)-1$$

**TABLE 3:** Summary table of initial in‐vivo dosimetry results for BeO for photons and electrons. Ranges listed are for one standard deviation.

Technique	%diff ‐or‐ cGy BeO vs. nanoDot	%diff BeO vs. Planned	%diff BeO vs. Commissioning (median)	Energy	Local tolerance
TBI—Lateral	1.72% ± 2.73% *N* = 9	+0.11% ± 4.42% *N* = 9	**–**	6 MV	±5%
TSI—Standing	**–**	+9.83% ± 9.70% *N* = 4, Thorax	−1.21% ± 12.42% *N* = 7	6 HDTSe	± 10%
TSI—Recumbent	4.31% ± 5.40% *N* = 9	+6.20% ± 4.76% *N* = 4, Thorax	−5.15% ± 12.7% *N* = 9	6 HDTSe	± 15%
Electron, en‐face	**–**	+1.69% ± 2.76% *N* = 6	**–**	6,9 MeV	± 5%
Pacemaker vs. nanoDot	3.52 cGy vs. 5.80 cGy 2.42 cGy vs. 3.70 cGy *N* = 2	**–**	**–**	Out‐of‐field dose	Per TG‐203

For our lateral TBI cases, we observed a mean difference of +1.72% ± 2.73% (*n* = 9) between BeO and Al_2_O_3_ (nanoDot) OSLs, and +0.11% ± 4.42% (*n* = 9) between BeO OSLs and expected plan dose. For clinical electron cases, a difference of +2.03% percent was observed in our singular comparison with nanoDots, while a mean difference of +1.69% ± 2.76% (*n* = 6) was seen when comparing against planned doses. For the recumbent TSET patient, a direct comparison with nanoDots was made. Here, the mean difference was 4.31% ± 5.40% (*n* = 9). Additionally, BeO TSET (recumbent and standing) measurements were compared against corresponding median commissioning values. Mean differences of ‐1.21% ± 12.42% (*n* = 7) and ‐5.15% ± 12.7% were calculated for the standing and recumbent techniques, respectively.

Regarding out‐of‐field pacemaker results, 8 measurements were made, 2 of which were performed side‐by‐side with nanoDot OSLs. For these 2 cases, located 7 and 15 cm from the field edge respectively, BeO readings were lower than Al_2_O_3_:C (3.52 cGy vs. 5.78 cGy, 2.42 cGy vs. 3.7 cGy) which makes sense in light of the difference in effective atomic number (7.2 vs. 11.3) and the lower mean energy of patient‐scattered radiation and head leakage^18^ which dominate far from the field edge. 3 out of the 6 remaining cases involved pacemakers in close proximity to the field edge (< 3 cm), and Eclipse‐calculated doses were used to judge the accuracy of these dosimeters. Measured doses for all 3 fell within the calculated dose range. The final 3 cases involved pacemakers located far from the field edge (> 15 cm). Although no reference was available for direct comparison, readings for these cases were judged to be consistent with historical measurements.

### 3.7 Average dose rate dependence

myOSLchip BeO elements showed no average dose‐rate dependence outside of the uncertainty of the measurement. For 6FFF, chip response between 1400 and 600 MU/min was 1.0022 ± 0.0026. For 10FFF—2400 vs. 400 MU/min—the measured difference was 1.0038 ± 0.0027. Finally, for 6 MV (100 vs. 600 MU/min), the signal difference was 0.998 ± 0.0019.

With respect to 6 HDTSe (DR = 2500 MU/min), an over‐response relative to 6 MV of 0.9805 ± 0.0042 was observed. It should be noted, however, that this is practically equal in magnitude to the beam quality correction factor measured for 6 MeV of 0.9812 ± 0.0061 reported in 3.2, indicating that the HDTSe over‐response is related purely to beam quality and not dose rate.

### 3.8 Angular dependence

**FIGURE 9:**
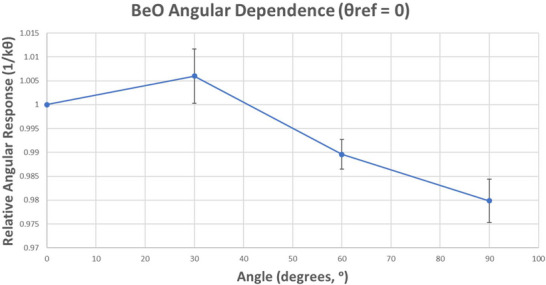
Relative angular response of myOSLchip dosimeters in a 6 MV beam. Response is normalized to 0 degrees. Error bars indicate one standard deviation (1σ).

Relative to 0°, a minor signal enhancement of 0.6% ± 0.57% was measured at 30°. Beyond this angle, the measured angular response declined to ‐1.04% ± 0.31% at 60° and ‐2.02% ± 0.45% at 90° as depicted in Figure 9 below.

### 3.9 Clinical operations and routine system quality assurance

In accordance with manufacturer recommendations, a photomultiplier tube (PMT) Dark Count limit of 30 counts and a Reference Light Counts limit of ± 5% around the empirically‐measured reader mean were entered into the reader software with enforcement configured on an “every reading” basis. The reference light counts value is derived from PMT response to a known test stimulus from the reader. During commissioning, a maximum Dark Count of 3 was observed and all reference light counts fell within 1.5% of the reader mean. As noted previously, PMT gain is adjusted between high and low dose regions. This impacts the reference light count as well, requiring an adjustment (of 4.16x) to the limiting range to avoid a read error for high dose readings.

Additionally, a set of Constancy OSLs was created for use in the performance of Daily QA by irradiating 3 chips to a known dose (2 Gy). Prior to performance of any clinical measurements, staff are required to readout at least one constancy chip and to compare the measured signal against the expected (based on depletion). A local tolerance of ± 5% is enforced.

## 4 DISCUSSION

Investigation of the myOSLchip system was motivated by the sudden, unexpected decision by Landauer to discontinue servicing and support for the microStar reader and sales of the nanoDot OSL dosimeters. Table 4 provides a comparative summary of dosimeter characteristics between BeO (myOSLchip, RadPro) and Al_2_O_3_:C (nanoDot, Landauer) from this study and the literature.

**TABLE 4:** A summary of measured and reported OSL dosimeter properties for BeO and Al_2_O_3_:C.

Property	BeO	Al_2_O_3_:C
Energy dependence	Relative to 6MV at d = 10 cm γ: ‐4.5% (2.5FFF) to +4.5% (15MV) e: +1.9% (6 MeV) to 4.3% (20 MeV)	Relative to 6 MV at d_max_; differing media γ: +0.5% (18 MV)^6^ +4.1% (18 MV)^21^ e: +1.9% (6‐20 MeV)^6^ µ = ‐3.6% (6‐22 MeV)^21^
Linearity	≤ 3% over 0.1 to 20 Gy	+2.5% at 2 Gy^19^ +15% at 10 Gy
Fading	+5% (t+5 min); +1% (t+15 min) relative to t+24 h −5% (t+100 d) relative to t+30 min^20^	−2.5% (t+2.5d) relative to t+10 min^2^ −1.8% (t+38d) relative to t+17d^21^
Depletion	µ = ‐2.1% ± 0.2% / read, all dose ranges	−2.0% (strong beam, low dose)^22^ −0.2% (weak beam, high dose)
Sensitivity change with accumulated dose	µ = ‐1.98% ± 0.55%, 0–15 Gy µ = ‐2.38% ± 0.85% (Δ = ‐0.5%), 15–32 Gy *15 Gy pre‐irradiation by vendor not included in dose ranges.	No detectable change up to 20 Gy; 4%/10 Gy (up to 50 Gy)^2^ +5% (1‐10 Gy); ‐18% at ∼80 Gy^5^
Angular dependence	−2% at 90 degrees relative to en‐face (6 MV)	−3% to ‐4% for 90 deg. relative to enface^23^ (6/18 MV) −2% for 90 deg. relative to en face**24**
Typical use profile	Reusable	Single use/disposable

The Duke Health Enterprise offers Radiation Oncology services at seven locations across eastern North Carolina and performs an average of 400 billable in‐vivo dose measurements per year. Therefore, in evaluating potential replacement systems, a balanced approach considering initial and enduring cost, reliability and accuracy, installation burden, and time requirements for commissioning and routine quality assurance was necessary. Furthermore, most in‐vivo dose measurements at Duke University Medical Center are performed in support of special treatment procedures (TBI, TSET), which imposes additional performance requirements on candidate systems.^16^, ^25^

Energy dependence (reference: 6 MV, depth = 10 cm) was observed to increase with increasing energy. This effect was observed for both photons and electrons, with variations of ‐4.5% (2.5FFF) to +4.5% (15 MV) for photons and +1.9% (6 MeV) to +4.3% (20 MeV) for electrons. Presently, there is little in the way of published results for comparison with respect to beryllium oxide dosimeters in the clinical setting. Analogous studies of aluminum oxide OSLs reported a wide range of results. Yukihara et al.^6^ measured a negligible energy dependence at d_max_ of +0.5% between 6 MV and 18 MV beams using a combination water/solid water phantom^6^ while Schembri et al.^21^ noted a ‐4.1% difference in response between the same photon beam energies using a polystyrene phantom, also at d_max_. There was similar disagreement in magnitude and sign when comparing electron beam responses (6 MeV to 20 MeV) to 6 MV in these studies, with Yukihara et al.^6^ reporting an average over‐response of 1.9% for high‐energy electrons and Schembri et al.^21^ measuring an average ‐3.6% under‐response. Jursinic et al.^2^ noted no variation in OSL energy dependence across the same range of electron energies and up to 15 MV for photons, while Ponmalar et al.^26^ measured decreasing over‐response relative to 6 MV with increasing electron energy over the range spanning 6 MeV (+2.0%) to 20 MeV (+0.9%). These varying results underscore the need for additional testing and comparison in order to build consensus for beryllium oxide (Thermalox 995) systems.

Superior linearity characteristics were observed for beryllium oxide as compared with aluminum oxide; however, this result was due to a combination of material‐based factors and hardware design choices by the system manufacturer. With respect to aluminum oxide, supralinear behavior with increasing dose is a well‐known attribute, with previous studies noting onset at doses as low as 2 Gy,^21^ growing to as much as 10%–20% at doses of 10 Gy.^2^, ^13^ These studies utilized microStar readers produced by Landauer, which varied signal strength at the PMT by changing the illumination intensity (strong or weak) incident upon the dosimeter and utilizing separate calibration curves for dose calculations. The cross‐over point for these devices was typically set quite low (∼10–15 cGy) such that the majority of dose levels evaluated were measured with fixed system settings. In the myOSLchip reader, the signal production and processing systems have been implemented in a notably different way. In this system, the cross‐over point is set much higher (∼2.25 × 10^5^ counts, or ∼12 Gy); further, instead of varying illumination intensity, PMT gain is reduced for high‐dose measurements. The net effect of this approach is a division of supralinear behavior into two regions—(0, 12 Gy) and [12 Gy, ∞)—and a maximum supralinear effect of ∼3% across the entire dose range from 0.1 to 20 Gy for this system. The average signal depletion value of 2.13%/reading is noteworthy for users previously accustomed to the microStar family of readers from Landauer. The microStar ii reader used a threshold of 15 cGy to divide low and high dose readings, employing a longer pulse width to the read LED for measurements below this threshold. This resulted in a differential depletion rate of 2.0% and 0.2% per reading for low dose and high dose measurements, respectively.^22^ For the original microStar reader, reported values were even lower, on the order of < 0.1%/reading.^2^, ^10^ Of particular interest, however, is the observation of a larger initial depletion value of 2.6%/reading. TG‐191 notes that this finding has been sporadically reported in the past for other unspecified systems, but does not offer further details. More investigation is needed to ascertain the validity of this finding.

With respect to angular dependence, TG191 notes a general trend in the literature of decreasing relative dosimeter signal with increasing angular deviation from an en face orientation for nanoDots. It is hypothesized that increased out‐scattering of electrons from the sensitive volume in an edge‐on orientation is responsible for this trend. Given the geometric nature of the problem and the difference in sensitive element shape between myOSLchip dosimeters (square) and nanoDots (discs), a direct comparison is complicated; however general response trends between the two systems appear to be comparable in terms of sign and magnitude. In our study, a slight signal *enhancement* relative to 0° of 0.6% was measured at an angle of 30° with subsequent reduction to ‐1% at 60° and ‐2% at 90°. In general, these results agree very well with nanoDot values reported in the literature. As cited within TG191, Lehmann et al^24^ measured a signal decline in a 6 MV beam of ‐0.5% at 30°, but nearly identical values at 60° and 90° using a custom OSL holder embedded within a CIRS phantom at a depth of 10 cm. These values were corroborated in their study via Monte Carlo simulations. Kerns et al.^23^ evaluated the angular dependence for nanoDots in high‐energy photon beams (6 and 18 MV) and observed a 3%–4% decrease in dosimeter response for OSLs positioned edge‐on toward the beam central axis relative to an en‐face orientation.

A notable limitation of this study is the lack of long‐term characterization of the spontaneous signal decay behavior (fading) of the dosimeter. While this paper only evaluates fading over the first 24 h post‐irradiation, other studies have provided strong evidence of good signal retention in the material. Bulur et al.^27^ evaluated fading behavior for Thermalox 995, noting signal stabilization after approximately 33 min with minimal subsequent loss out to 3 h. Sommer^20^ noted a 6% signal loss for Thermalox 995 over 6 months when normalized to 30 min post‐irradiation. These values compare well against aluminum oxide OSLs with Mrčela et al. noting a 4% signal loss relative to a 1 h post‐irradiation reference. This remains an area of active research within our clinic and we expect to present the results of this analysis in a future study.

Finally, a note regarding dose rate dependence. Linear accelerator (LINAC) dose rate is predominantly controlled by altering pulse repetition frequency in the accelerating waveguide.^2^ Therefore, users only possess the ability to vary the average dose rate (in terms of MU/min), not the instantaneous dose rate (dose per pulse). Acknowledging that limitation, Jursinic et al.^2^ did study the effect of varying instantaneous dose rate on aluminum oxide dosimeters and observed no sensitivity variation outside of the measurement uncertainty for dose‐per‐pulse values ranging from 0.22 Gy/s up to 95.6 Gy/s.

## 5 CONCLUSION

As demonstrated in this paper, the myOSLchip system is capable of producing precise and accurate in‐vivo dosimetric results across a wide range of clinical modalities and techniques (Table 3) when commissioned and tested in accordance with the framework and methodology presented in TG191. The beryllium oxide dosimeters utilized by the system demonstrate excellent linearity with dose and minimal energy dependence across a wide range of clinical dose levels and beam energies (Table 4). Additionally, chip size, portability, and ease of use make them ideal measurement devices in multi‐site, high throughput settings, particularly where multi‐point measurements are common. Finally, their rapid signal stabilization and quick readout times allow for these accurate results to be obtained on timelines well‐suited to support clinical operations.

## AUTHOR CONTRIBUTIONS

Dr Joseph P. Kowalski: Test design/Methodology, data acquisition, data analysis, initial paper draft. Brett G. Erickson, MS: Test design/Methodology, data acquisition, data analysis, draft paper revisions. Dr Qiuwen Wu: Test design/Methodology, data analysis, draft paper revisions. Dr Xinyi Li: Data acquisition, data analysis, draft paper revisions. Dr Sua Yoo: Data analysis, draft paper revisions.

## CONFLICT OF INTEREST STATEMENT

The authors declare no conflicts of interest.
